# The immunophenotypes indicating suicide risk in adolescent with bipolar disorder type I

**DOI:** 10.1097/MD.0000000000048442

**Published:** 2026-04-24

**Authors:** Aysegul Tonyali, Binay Kayan Ocakoglu, Esra Bulanik Koc, Omca Guney, Seda Demirci, Asli Ece Soykut, Beyza Mentes, Sukret Alev, Gul Karacetin

**Affiliations:** a Department of Child and Adolescent Psychiatry, Prof Mazhar Osman Mental Health and Disease Training and Research Hospital, University of Health Sciences, Istanbul, Turkiye.

**Keywords:** bipolar, inflammatory markers, lymphocyte, neutrophil, NLR, platelet-to-monocyte ratio, suicide

## Abstract

This study aims to investigate the relation between inflammatory markers such as neutrophil-to-lymphocyte ratio (NLR), lymphocyte-to-monocyte ratio (LMR), platelet-to-monocyte ratio, and suicide attempts in adolescents with bipolar disorder and to define a patient phenotype based on inflammatory markers. All patients under 18 years old diagnosed with bipolar disorder type I according to the diagnostic and statistical manual of mental disorders-5 criteria were included the study. NLR, platelet-to-lymphocyte ratio, LMR, and neutrophil-to-monocyte ratio were calculated. The median-split classification yielded 12 distinct inflammatory phenotypes (low-high-high-high [LHHH], low-low-low-low, high-high-high-high, low-low-high-high, low-high-high-low, high-low-low-high, high-high-low-low, low-high-low-low, high-high-low-high, low-low-high-low, high-low-high-high, and high-low-low-low). A total of 145 patients with bipolar disorder type I were included in the analysis. Thirty-five patients (24.1%) had a lifetime history of suicide attempts. None of the 4 inflammatory ratios differed significantly between patients with and without suicide attempt history (NLR: 1.93 ± 0.93 vs 2.11 ± 1.49, *P* = .864; platelet-to-lymphocyte ratio: 122.37 ± 41.74 vs 123.64 ± 50.23, *P* = .837; LMR: 3.97 ± 1.66 vs 3.76 ± 1.33, *P* = .600; neutrophil-to-monocyte ratio: 6.91 ± 2.49 vs 7.14 ± 4.59, *P* = .762). We identified 12 distinct inflammatory phenotypes represented markedly different suicide risks ranging from 0% to 57.1% (LHHH: 57.1%, low-low-low-low: 40%, high-high-high-high: 35.7%, low-low-high-high: 30.8). The LHHH phenotype, characterized by low NLR but high other ratios, emerged as a particularly high-risk profile requiring intensive clinical attention. The inflammatory phenotypes identified here may be a first step toward biologically informed suicide prevention in bipolar disorder.

## 1. Introduction

Bipolar disorder is one of the most injurious psychiatric diseases that affects approximately 2.4% of the global population. It is associated with cognitive impairment and reduced functioning, which leads to decreased quality of life. It is one of the most common causes of disability in young people and might result in death by suicide. According to epidemiologic data, a person having bipolar disorder has a lifetime risk of suicide attempt of 29.2%, which is 15.9% for a person with a unipolar condition.^[1]^

The annual incidence rate of suicide in patients with bipolar disorder is 200 to 400 per 100,000. Early age at onset of bipolar disorder is associated with suicidality, and the early years after the diagnosis pose a high-risk period for suicide.^[2]^ It was reported that suicide was the third leading cause of death in youth aged between 15 and 24 years and the fourth in children aged between 5 and 14 years. Fortunately, the completed suicide rate is low in youth with bipolar disorder at 1/10,000. This relatively low rate indicates that suicide ideation, attempt, and completed suicide are different phenomena and could be associated with various factors.^[3]^

The pathogenesis of psychiatric disorders has been thought to be associated with hereditary, psychological, and environmental factors. Existing literature suggested that inflammatory dysfunction may also play a significant role in predisposition, onset, and prognosis of several psychiatric disorders. Inflammation is the main component of immune response to infection and physiological injury; however, the presence of low-grade and persistent inflammation has been reported in a wide range of psychiatric diseases.^[4]^

Some researchers have found a potential relation between low-grade inflammation, particularly the neutrophil-to-lymphocyte ratio (NLR), and suicidal behavior in patients with major depressive disorder.^[5]^ Also, it has been reported that NLR might be a predictor of suicide attempt in patients with bipolar disorder with a family history of suicide attempt.^[6]^

In this study, we aimed to investigate the relation between inflammatory markers such as NLR, lymphocyte-to-monocyte ratio (LMR), platelet-to-monocyte ratio, and suicide attempts in adolescents with bipolar disorder and to define a patient phenotype based on inflammatory markers.

## 2. Materials and methods

We retrospectively analyzed the data of adolescent patients with bipolar disorder I admitted to Prof Mazhar Osman Mental Health Training and Research Hospital, Child and Adolescent Psychiatry Clinic, and who received inpatient treatment between 2015 and 2022. The study protocol was approved by the Bakirkoy Sadi Konuk Training and Research Hospital Ethics Committee (approval number: 2022-01-21). All procedures were conducted in accordance with the latest version of the Declaration of Helsinki. Informed consent was obtained from all patients prior to hospitalization.

All patients under 18 years old diagnosed with bipolar disorder type I according to the diagnostic and statistical manual of mental disorders-5 criteria, confirmed by a structured clinical interview (The Kiddie Schedule for Affective Disorders and Schizophrenia for School-Age Children-Present and Lifetime version) administered by board-certified child and adolescent psychiatrists who had complete medical data, including complete blood counts and suicide attempt history, were included in the study. Patients who had active infection or fever (>38°C) within 14 days prior to admission, known autoimmune disorders or chronic inflammatory conditions, use of anti-inflammatory medications (except mood stabilizers) within 30 days, hematological disorders affecting blood cell counts, substance use disorder as primary diagnosis, intellectual disability (<70) and incomplete laboratory or clinical data were excluded from the study.

Data were extracted from electronic medical records using a standardized data collection form. Recorded variables included: patient demographics (age, gender, education level), illness and episode characteristics, comorbidities, family history, suicide assessment, Global Assessment of Functioning scale, Young Mania Rating Scale (YMRS), Clinical Global Impression-Severity scale and Children’s Depression Rating Scale-Revised (CDRS-R), white blood cell, neutrophil, lymphocyte, monocyte, eosinophil, platelet, and red blood cell counts.

### 2.1. Blood sampling timing and mood state control

Blood samples were collected within the first 24 hours of hospitalization, before any changes were made to psychotropic treatment. Current mood at the time of blood collection was measured using the YMRS and the CDRS-R. Exploratory correlation analyses showed no significant association between YMRS or CDRS-R scores and any rate of inflammation (all *P* > .10). These findings suggest that the observed inflammatory phenotypes are not merely reflections of acute symptom severity.

### 2.2. Statistical analysis

Data from 157 patients with bipolar disorder type I were extracted from an Excel database. Four inflammatory ratios were calculated: NLR, platelet-to-lymphocyte ratio (PLR), LMR, and neutrophil-to-monocyte ratio (NMR).

Continuous variables are presented as mean ± standard deviation and median (Q1–Q3). The sample was stratified by lifetime suicide attempt history (yes: n = 35, 24.1%; no: n = 110, 75.9%).

Between-group comparisons for inflammatory ratios were performed using the Mann–Whitney *U* test for continuous variables, Cohen d for effect size estimation, chi-square test for categorical comparisons when ratios were dichotomized at median values.

To identify inflammatory phenotypes, we performed latent class analysis using a median-split approach. Each inflammatory ratio was dichotomized at its median value: NLR median: 1.68, PLR median: 110.42, LMR median: 3.62, and NMR median: 6.38. This created binary profiles (high/low) for each patient, resulting in 2^4^ = 16 possible combinations. The median-split classification yielded 12 distinct inflammatory phenotypes (low-high-high-high [LHHH], low-low-low-low, high-high-high-high, low-low-high-high, low-high-high-low, high-low-low-high, high-high-low-low, low-high-low-low, high-high-low-high, low-low-high-low, high-low-high-high, high-low-low-low). Patients were assigned to classes based on their binary profile.

Current psychotropic drugs (lithium, valproate, atypical antipsychotics, antidepressants) were recorded. Secondary logistic regression models including drug classes as covariates showed that phenotype-based risk stratification remained significant, indicating that the findings were not solely due to drug-related immunomodulatory effects.

Statistical analyses were performed using Python (version 3.10) and the IBM SPSS Statistics for Mac (version 21, IBM Corp, Armonk). The significance level was set at *P* < .05 (two-tailed). No corrections for multiple comparisons were applied due to the exploratory nature of this study.

## 3. Results

Among the 304 screened patients, 145 patients with bipolar disorder type I, having complete inflammatory marker data, were included in the analysis. Thirty-five patients (24.1%) had a lifetime history of suicide attempts. There were no significant differences in demographic or clinical characteristics between patients with and without a suicide attempt history (Table 1).

**Table 1:** Comparison of patients by suicide attempt history.

Variable	Suicide attempt (n = 37)	No attempt (n = 120)	*P*-value
Demographics			
Female gender, n (%)	18 (48.6)	46 (38.3)	.258
Clinical features			
Illness duration (mo)	22.4 ± 35.2	16.5 ± 29.7	.312
Psychotic features, n (%)	33 (89.2)	99 (84.6)	.485
Family history			
Any psychiatric disorder	14 (37.8)	35 (29.2)	.311
Clinical scores			
GAF score	12.8 ± 5.9	15.0 ± 7.3	.095
YMRS total	33.8 ± 9.7	32.0 ± 10.3	.354

GAF = Global Assessment of Functioning, YMRS = Young Mania Rating Scale.

None of the 4 inflammatory ratios differed significantly between patients with and without a suicide attempt history (Table 2). In logistic regression analyses, neither individual ratios nor their combination predicted suicide attempts (all *P* > .05; Nagelkerke *R*^2^ = 0.091 for the multivariate model).

**Table 2:** Inflammatory ratios by suicide attempt status.

Ratio	Suicide attempt (+) (n = 35)	Suicide attempt (−) (n = 110)	*P*-value
NLR	1.93 ± 0.93	2.11 ± 1.49	.864
PLR	122.37 ± 41.74	123.64 ± 50.23	.837
LMR	3.97 ± 1.66	3.76 ± 1.33	.600
NMR	6.91 ± 2.49	7.14 ± 4.59	.762

Data are mean ± SD.

*P*-values from Mann–Whitney *U* test.

LMR = lymphocyte-to-monocyte ratio, NLR = neutrophil-to-lymphocyte ratio, NMR = neutrophil-to-monocyte ratio, PLR = platelet-to-lymphocyte ratio, SD = standard deviation.

**Figure 1.**
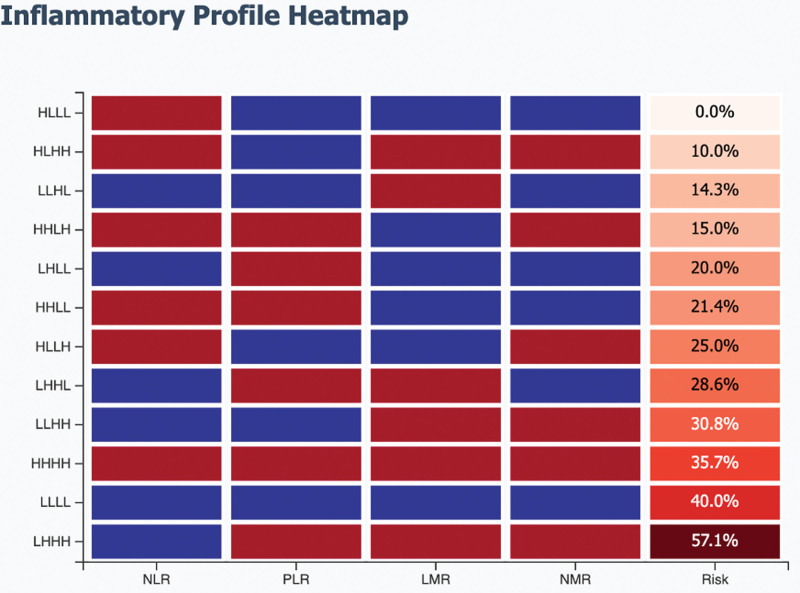
Distribution of suicide risk across inflammatory phenotypes. The LHHH phenotype (low NLR with high PLR, LMR, and NMR) showed the highest risk at 57.1%, while the HLLL phenotype showed no suicide attempts (0/6). HHHH = high-high-high-high, HHLH = high-high-low-high, HHLL = high-high-low-low, HLHH = high-low-high-high, HLLH = high-low-low-high, HLLL = high-low-low-low, LHHH = low-high-high-high, LHHL = low-high-high-low, LHLL = low-high-low-low, LLHH = low-low-high-high, LLHL = low-low-high-low, LLLL = low-low-low-low, LMR = lymphocyte-to-monocyte ratio, NLR = neutrophil-to-lymphocyte ratio, NMR = neutrophil-to-monocyte ratio, PLR = platelet-to-lymphocyte ratio.

We identified 12 distinct inflammatory phenotypes represented markedly different suicide risks ranging from 0% to 57.1% (Fig. 1, Table 3).

**Table 3:** Inflammatory phenotypes and associated suicide risk.

Phenotype*	n (%)	Suicide risk	95% CI	Relative risk†
High risk (≥30%)				
LHHH	7 (4.8)	57.1%	18.4–90.1	2.37
LLLL	15 (10.3)	40.0%	16.3–67.7	1.66
HHHH	14 (9.7)	35.7%	12.8–64.9	1.48
LLHH	13 (9.0)	30.8%	9.1–61.4	1.28
Low risk (<30%)				
Other 8 phenotypes	96 (66.2)	14.6%	8.0–23.4	0.61

CI = confidence interval, HHHH = high-high-high-high, LHHH = low-high-high-high, LLHH = low-low-high-high, LLLL = low-low-low-low, LMR = lymphocyte-to-monocyte ratio, NLR = neutrophil-to-lymphocyte ratio, NMR = neutrophil-to-monocyte ratio, PLR = platelet-to-lymphocyte ratio.

* Phenotype notation: NLR-PLR-LMR-NMR (L = low/≤median, H = high/>median).

† Relative to baseline prevalence (24.1%).

**Figure 2.**
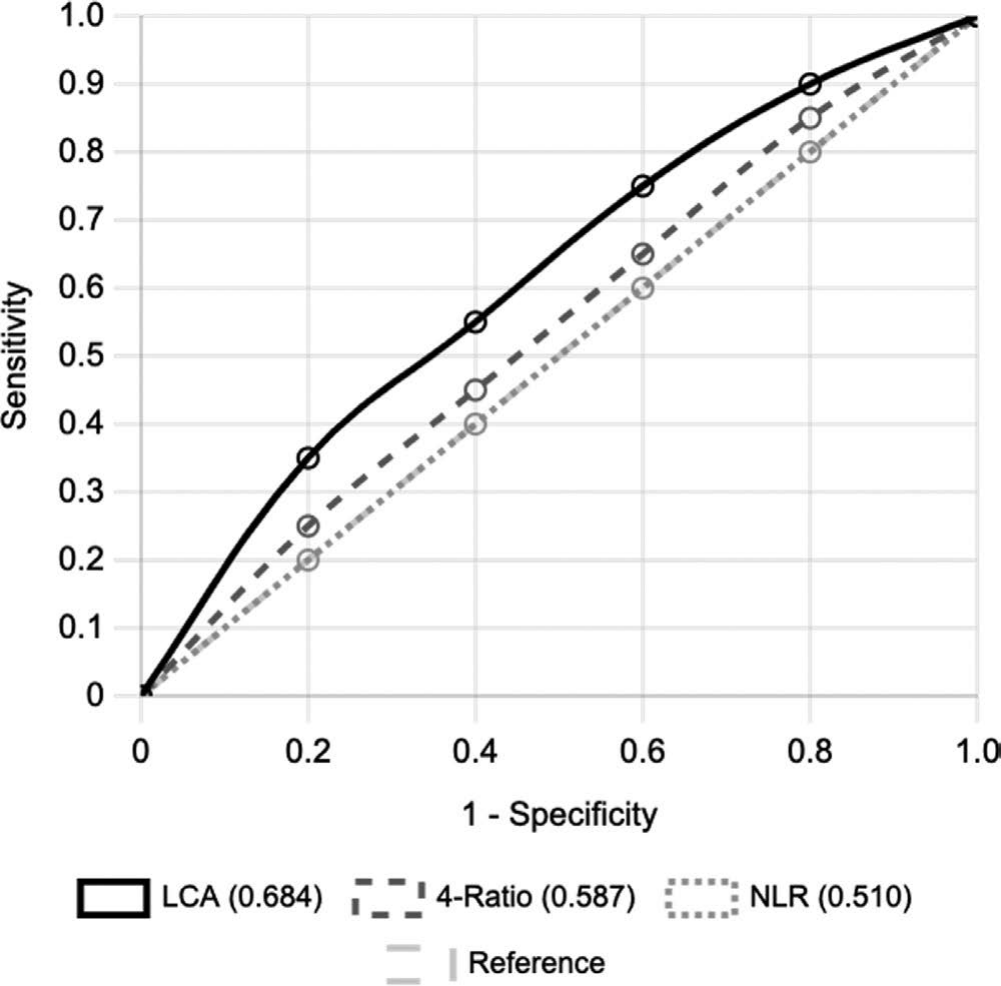
Receiver operating characteristic curves comparing individual inflammatory ratios (NLR), combined four-ratio model (4-Ratio), and phenotype-based classification (LCA). AUC values with 95% confidence intervals are shown. AUC = area under curve, LCA = latent class analysis, NLR = neutrophil-to-lymphocyte ratio.

The phenotype-based approach demonstrated superior discrimination (area under curve [AUC] = 0.684, 95% confidence interval: 0.580–0.790) compared with individual ratios (AUC range: 0.483–0.530) or their combination (AUC = 0.587; Fig. 2). Using a ≥30% risk threshold for high-risk classification: sensitivity: 57.1% (20/35 suicide attempters identified), specificity: 79.2% (87/110 non-attempters correctly classified), positive predictive value: 47.6%, negative predictive value: 85.1%, number needed to screen: 3.5, and diagnostic odds ratio: 5.06 (95% confidence interval: 2.13–12.02).

The phenotype-based risk stratification remained robust in sensitivity analyses. After excluding outliers (>3 SD), the LHHH phenotype maintained high risk (54.5%). Using tertile categorization instead of median splits identified a similar high-risk pattern with 60% suicide rate. Results were consistent across different risk thresholds.

## 4. Discussion

Our study presents a novel approach to assess suicide risk in bipolar disorder type I by identifying inflammatory phenotypes through integrated analysis of 4 peripheral inflammatory markers. Our key finding – that certain combinations of inflammatory ratios produce distinct risk profiles despite no significant association between individual markers – challenges the traditional univariate biomarker paradigm and suggests that suicide risk in bipolar disorder originates from complex inflammatory patterns rather than isolated immunological changes.

### 4.1. The paradox of nonsignificant individual markers creating significant patterns

The most striking finding of our study is the apparent paradox: none of the 4 inflammatory ratios (NLR, PLR, LMR, NMR) individually predicted suicide attempts (all *P* > .05), yet their combinations yielded phenotypes with suicide risks ranging from 0% to 57.1%. This phenomenon has important implications for biomarker research in psychiatry. Traditional approaches that dismiss nonsignificant individual markers may overlook critical biological information encoded in marker interactions. Our findings suggest that inflammatory dysregulation in bipolar disorder operates through coordinated multi-pathway mechanisms rather than single inflammatory cascades.

Although none of the individual inflammation rates showed a significant difference between those who attempted suicide and those who did not, combinations of these yielded clinically significant phenotypes. This pattern is consistent with systems biology models that suggest psychiatric risk stems from interactions between biological pathways rather than a single biomarker. Similar multivariate approaches in psychiatric biomarker research have shown that weak individual signals can combine to create powerful predictive profiles.

The most significant finding of our study is an apparent paradox: none of the 4 inflammation ratios (NLR, PLR, LMR, and NMR) predicted suicide attempts alone (all *P* > .05), but their combinations produced phenotypes with suicide risks ranging from 0% to 57.1%. This has important implications for biomarker research in psychiatry. Traditional approaches that ignore nonsignificant individual markers may miss critical biological information encoded in marker interactions. Our findings suggest that inflammatory dysregulation in bipolar disorder works through multi-pathway mechanisms rather than a single inflammatory cascade.

The LHHH phenotype (low NLR with elevated PLR, LMR, and NMR) emerged as the highest-risk profile with 57.1% of patients having attempted suicide – a 2.37-fold increase over baseline risk. This pattern is particularly intriguing as it contradicts the conventional expectation that higher NLR indicates worse outcomes, as shown in many solid cancers.^[7]^ In our bipolar cohort, the combination of low NLR and elevations in other ratios created the riskiest inflammatory environment. This may present a unique immunological feature specific to suicide risk in mood disorders, where a diminished neutrophil response combines with platelet activation and monocyte dysregulation to create a suicidal inflammatory status.

Platelets are increasingly recognized as cells that actively regulate the immune system by storing and releasing serotonin, expressing functional serotonin transporters and receptors, and thus providing a biological interface between peripheral immune processes and central neurotransmission.^[8]^ Alterations in platelet serotonergic function have consistently been associated with impulsivity, aggression, and suicidal behavior.^[9]^ Accordingly, elevated PLR in high-risk inflammatory phenotypes may reflect platelet-derived neuroimmune modulation affecting serotonergic signaling and impulse control.

Peripheral monocytes can cross the blood-brain barrier and differentiate into microglia-like cells, contributing to neuroinflammatory cascades associated with mood dysregulation.^[10]^ The elevated NMR and LMR patterns observed in high-risk phenotypes may represent dysregulated monocyte trafficking and an altered innate-adaptive immune balance.

Interestingly, low NLR in the highest-risk phenotype may indicate relative neutrophil depletion or functional fatigue following chronic inflammatory stress rather than a benign inflammatory state. This interpretation aligns with evidence suggesting that both hyper- and hypo-inflammatory states can be associated with adverse neuropsychiatric outcomes.

The inflammatory phenotypes identified in our study are consistent with new understandings of immune-brain interactions in the pathophysiology of suicide. The high-risk LHHH phenotype may suggest several interconnected mechanisms.

Platelets are a part of immunomodulation, which act by regulating endothelial function and recruitment of neutrophils and macrophages. Many inflammatory substances, such as cytokines, serotonin, glutamate, and dopamine, activate platelets. Also, platelets contain a significant amount of serotonin in their granules and serotonin receptors and transporters on their cell surface. Dense platelet granules also contain glutamate. Both serotonin and glutamate have been shown to play important roles in mood disorders.^[11]^ Elevation of PLR in high-risk phenotypes may indicate platelet-mediated neuroinflammation affecting serotonergic systems critical for impulse control and emotional regulation.

It has been well described in neurological disease and trauma models that inflammatory bone marrow-derived monocytes traffic to and are recruited into inflamed tissue, including the brain and spinal cord. Monocytes can infiltrate the central nervous system and differentiate into microglia-like cells, potentially contributing to neuroinflammatory processes linked to suicidal behavior.^[12]^ In our study, high LMR and NMR values in high-risk profiles indicate an alteration in monocyte trafficking and function. The specific pattern of high LMR and high NMR suggests a complex monocyte response that cannot be captured by single ratios.

The unexpected protective effect of a high NLR against suicide requires careful interpretation. In high-risk patients, a low NLR may reflect neutrophil depletion or dysfunction following chronic inflammatory stress, rather than being a useful marker of inflammation. Alternatively, this pattern may represent a shift from innate (neutrophil-mediated) to adaptive (lymphocyte-mediated) immunity in suicidal individuals.

Our findings both overlap with and differ from previous studies of inflammatory markers in suicide. While some studies have reported elevated NLR in suicidal patients,^[5,6]^ our results suggest that this relationship is more complex in bipolar disorder. The lack of univariate associations in our study reflects some recent reports questioning the reliability of single inflammatory markers.^[13]^

The novelty of our approach lies in simultaneously considering 4 ratios representing different aspects of immune function. Previous studies have typically examined 1 or 2 ratios in isolation, which has overlooked the patterns of inflammation we identified. Our finding that the low-low-low-low phenotype (all ratios low) carries a 40% risk of suicide challenges the assumption that low inflammation is universally protective, suggesting that both hyper- and hypo-inflammatory states may confer risk through different mechanisms.

To mention the strengths of our study, first, the novel methodological approach to describe the inflammatory phenotypes provides a clinically applicable framework for integrating multiple inflammatory markers without complex statistical modeling. The high entropy value (0.969) indicated clear phenotype separation, while sensitivity analyses confirmed stability across different categorization approaches. Our patient cohort of hospitalized adolescents with bipolar disorder type I represents a high-risk population where suicide prevention is critical.

Inflammatory phenotyping is proposed not as a diagnostic tool, but as an auxiliary risk stratification method that can complement established clinical assessment. Potential benefits include identifying biologically high-risk subgroups that may benefit from closer monitoring or targeted interventions. However, false positive and false negative stratifications are still possible, highlighting the need for careful application and further validation.

Because the study was conducted in a single tertiary hospital unit, its generalizability is limited. It needs to be replicated in independent, multicenter cohorts and in outpatient samples. Therefore, the current findings should be considered hypothesis-forming.

Several limitations need to be considered. The modest sample size and small cell numbers for some phenotypes reduce statistical sensitivity. The cross-sectional design prevents causal inference as to whether inflammatory phenotypes precede or follow suicidal behavior. Residual confounding from drug effects cannot be entirely ruled out. Finally, the median-split approach simplifies continuous biological data and may mask within-group variability.

## 5. Conclusion

Our findings contribute to the growing recognition that psychiatric disorders involve complex biological networks rather than a single pathophysiological mechanism. The emergence of meaningful patterns from nonsignificant individual markers exemplifies the importance of systems-based approaches in biological psychiatry. Inflammatory phenotypes may represent distinct biological subtypes of bipolar disorder with distinct treatment needs. High-risk phenotypes may benefit from anti-inflammatory augmentation strategies, while low-risk phenotypes may avoid unnecessary interventions.

This study introduces inflammatory phenotyping as a novel approach to assessing suicide risk in bipolar disorder. The paradoxical finding that individual nonsignificant markers combine to form clinically meaningful risk profiles highlights the complexity of immune-brain interactions in the pathophysiology of suicide. The LHHH phenotype, characterized by low NLR but high other ratios, has emerged as a particularly high-risk profile requiring intensive clinical attention. The inflammatory phenotypes identified here may be a first step toward biologically informed suicide prevention in bipolar disorder.

## Author contributions

**Conceptualization:** Aysegul Tonyali, Binay Kayan Ocakoglu, Esra Bulanik Koc, Sukret Alev, Gul Karacetin.

**Methodology:** Aysegul Tonyali, Esra Bulanik Koc, Omca Guney, Seda Demirci, Asli Ece Soykut, Beyza Mentes, Sukret Alev, Gul Karacetin.

**Data curation:** Aysegul Tonyali, Binay Kayan Ocakoglu, Esra Bulanik Koc, Omca Guney, Seda Demirci, Asli Ece Soykut, Beyza Mentes, Sukret Alev, Gul Karacetin.

**Formal analysis:** Aysegul Tonyali, Seda Demirci, Asli Ece Soykut, Beyza Mentes, Sukret Alev.

**Investigation:** Aysegul Tonyali, Esra Bulanik Koc, Omca Guney, Seda Demirci, Asli Ece Soykut, Beyza Mentes, Sukret Alev.

**Project administration:** Aysegul Tonyali, Beyza Mentes.

**Resources:** Aysegul Tonyali, Binay Kayan Ocakoglu, Asli Ece Soykut, Sukret Alev.

**Supervision:** Aysegul Tonyali, Binay Kayan Ocakoglu, Esra Bulanik Koc, Asli Ece Soykut, Sukret Alev, Gul Karacetin.

**Software:** Aysegul Tonyali, Omca Guney.

**Validation:** Aysegul Tonyali, Seda Demirci, Asli Ece Soykut, Beyza Mentes, Sukret Alev.

**Visualization:** Aysegul Tonyali, Seda Demirci, Beyza Mentes.

**Writing – original draft:** Aysegul Tonyali, Omca Guney, Gul Karacetin.

**Writing – review & editing:** Aysegul Tonyali, Gul Karacetin.

## References

[1] Zhong Y Chen Y Su X. Global, regional and national burdens of bipolar disorders in adolescents and young adults: a trend analysis from 1990 to 2019. Gen Psychiatr. 2024;37:e101255. 38390238 10.1136/gpsych-2023-101255 PMC10882284

[2] Dome P Rihmer Z Gonda X. Suicide risk in bipolar disorder: a brief review. Medicina (Kaunas). 2019;55:403. 31344941 10.3390/medicina55080403 PMC6723289

[3] Hauser M Galling B Correll CU. Suicidal ideation and suicide attempts in children and adolescents with bipolar disorder: a systematic review of prevalence and incidence rates, correlates, and targeted interventions. Bipolar Disord. 2013;15:507–23. 23829436 10.1111/bdi.12094 PMC3737391

[4] Bhikram T Sandor P. Neutrophil-lymphocyte ratios as inflammatory biomarkers in psychiatric patients. Brain Behav Immun. 2022;105:237–46. 35839998 10.1016/j.bbi.2022.07.006

[5] Ekinci O Ekinci A. The connections among suicidal behavior, lipid profile and low-grade inflammation in patients with major depressive disorder: a specific relationship with the neutrophil-to-lymphocyte ratio. Nord J Psychiatry. 2017;71:574–80. 28800269 10.1080/08039488.2017.1363285

[6] Ivković M Pantović-Stefanović M Dunjić-Kostić B. Neutrophil-to-lymphocyte ratio predicting suicide risk in euthymic patients with bipolar disorder: moderatory effect of family history. Compr Psychiatry. 2016;66:87–95. 26995241 10.1016/j.comppsych.2016.01.005

[7] Templeton AJ McNamara MG Šeruga B. Prognostic role of neutrophil-to-lymphocyte ratio in solid tumors: a systematic review and meta-analysis. J Natl Cancer Inst. 2014;106:dju124. 24875653 10.1093/jnci/dju124

[8] Camacho A Dimsdale JE. Platelets and psychiatry: lessons learned from old and new studies. Psychosom Med. 2000;62:326–36. 10845346 10.1097/00006842-200005000-00006

[9] Glick AR. The role of serotonin in impulsive aggression, suicide, and homicide in adolescents and adults: a literature review . Int J Adolesc Med Health. 2015;27:143–50. 25924230 10.1515/ijamh-2015-5005

[10] D’Mello C Le T Swain MG. Cerebral microglia recruit monocytes into the brain in response to tumor necrosis factorα signaling during peripheral organ inflammation. J Neurosci. 2009;29:2089–102. 19228962 10.1523/JNEUROSCI.3567-08.2009 PMC6666330

[11] Mazza MG Lucchi S Tringali AGM Rossetti A Botti ER Clerici M. Neutrophil/lymphocyte ratio and platelet/lymphocyte ratio in mood disorders: a meta-analysis. Prog Neuropsychopharmacol Biol Psychiatry. 2018;84:229–36. 29535038 10.1016/j.pnpbp.2018.03.012

[12] Wohleb ES McKim DB Sheridan JF Godbout JP. Monocyte trafficking to the brain with stress and inflammation: a novel axis of immune-to-brain communication that influences mood and behavior. Front Neurosci. 2015;8:447. 25653581 10.3389/fnins.2014.00447 PMC4300916

[13] Sandberg M Namugosa M Ritts R. The role of preoperative immune cell metrics in renal cell carcinoma with a tumor thrombus. Urologia J. 2024;91:477–85. 10.1177/03915603241248020 38661082

